# Supplementary material for: Hypomyelinating Leukodystrophy 15 (HLD15)-Associated Mutation of EPRS1 Leads to Its Polymeric Aggregation in Rab7-Positive Vesicle Structures, Inhibiting Oligodendroglial Cell Morphological Differentiation
Source: Polymers (Basel). 2021 Mar 29;13(7):1074. doi: 10.3390/polym13071074 (PMC8037150; doi:10.3390/polym13071074)
Supplement: Supplementary file 1 [file polymers-13-01074-s001.zip › Supplemental/EPRS Figure legends (supplemental).docx]

**Supplemental figure legends**

**Figure S1. Wild-type EPRS1 proteins are not co-localized with the ER marker (KDEL).** (A, B) FBD-102b cells were transfected with the plasmid encoding the wild-type construct of FLAG-tagged EPRS1 and stained with antibodies against the FLAG antigen (green) and the ER marker KDEL antigen (red). Scan plots were constructed along the white dotted lines in the direction of the arrows in the color images (green and red). Graphs showing the fluorescence intensities (arbitrary units) along the white lines in the direction of the arrows can be seen in the bottom panels.

**Figure S2. Wild-type EPRS1 proteins are not co-localized with the Golgi body marker (GM130).** (A, B) FBD-102b cells were transfected with the plasmid encoding the wild-type construct of FLAG-tagged EPRS1 and stained with antibodies against the FLAG antigen (green) and the Golgi body marker GM130 antigen (red). Scan plots were constructed along the white dotted lines in the direction of the arrows in the color images (green and red). Graphs showing the fluorescence intensities (arbitrary units) along the white lines in the direction of the arrows can be seen in the bottom panels.

**Figure S3. Wild-type EPRS1 proteins are not co-localized with the lysosome marker (LAMP1).** (A, B) FBD-102b cells were transfected with the plasmid encoding the wild-type construct of FLAG-tagged EPRS1 and stained with antibodies against the FLAG antigen (green) and the lysosome marker LAMP1 antigen (red). Scan plots were constructed along the white dotted lines in the direction of the arrows in the color images (green and red). Graphs showing the fluorescence intensities (arbitrary units) along the white lines in the direction of the arrows can be seen in the bottom panels.

**Figure S4. Wild-type EPRS1 proteins are not co-localized with the early endosome marker (Rab7).** (A, B) FBD-102b cells were transfected with the plasmid encoding the wild-type construct of FLAG-tagged EPRS1 and stained with antibodies against the FLAG antigen (green) and the early endosome marker Rab7 antigen (red). Scan plots were constructed along the white dotted lines in the direction of the arrows in the color images (green and red). Graphs showing the fluorescence intensities (arbitrary units) along the white lines in the direction of the arrows can be seen in the bottom panels.

**Figure S5. Wild-type EPRS1 proteins are not co-localized with another early endosome marker (Rab9).** (A, B) FBD-102b cells were transfected with the plasmid encoding the wild-type construct of FLAG-tagged EPRS1 and stained with antibodies against the FLAG antigen (green) and another early endosome marker Rab9 antigen (red). Scan plots were constructed along the white dotted lines in the direction of the arrows in the color images (green and red). Graphs showing the fluorescence intensities (arbitrary units) along the white lines in the direction of the arrows can be seen in the bottom panels.
