# Supplementary figures and images for: Hypomyelinating Leukodystrophy 15 (HLD15)-Associated Mutation of EPRS1 Leads to Its Polymeric Aggregation in Rab7-Positive Vesicle Structures, Inhibiting Oligodendroglial Cell Morphological Differentiation
Source: Polymers (Basel). 2021 Mar 29;13(7):1074. doi: 10.3390/polym13071074 (PMC8037150; doi:10.3390/polym13071074)

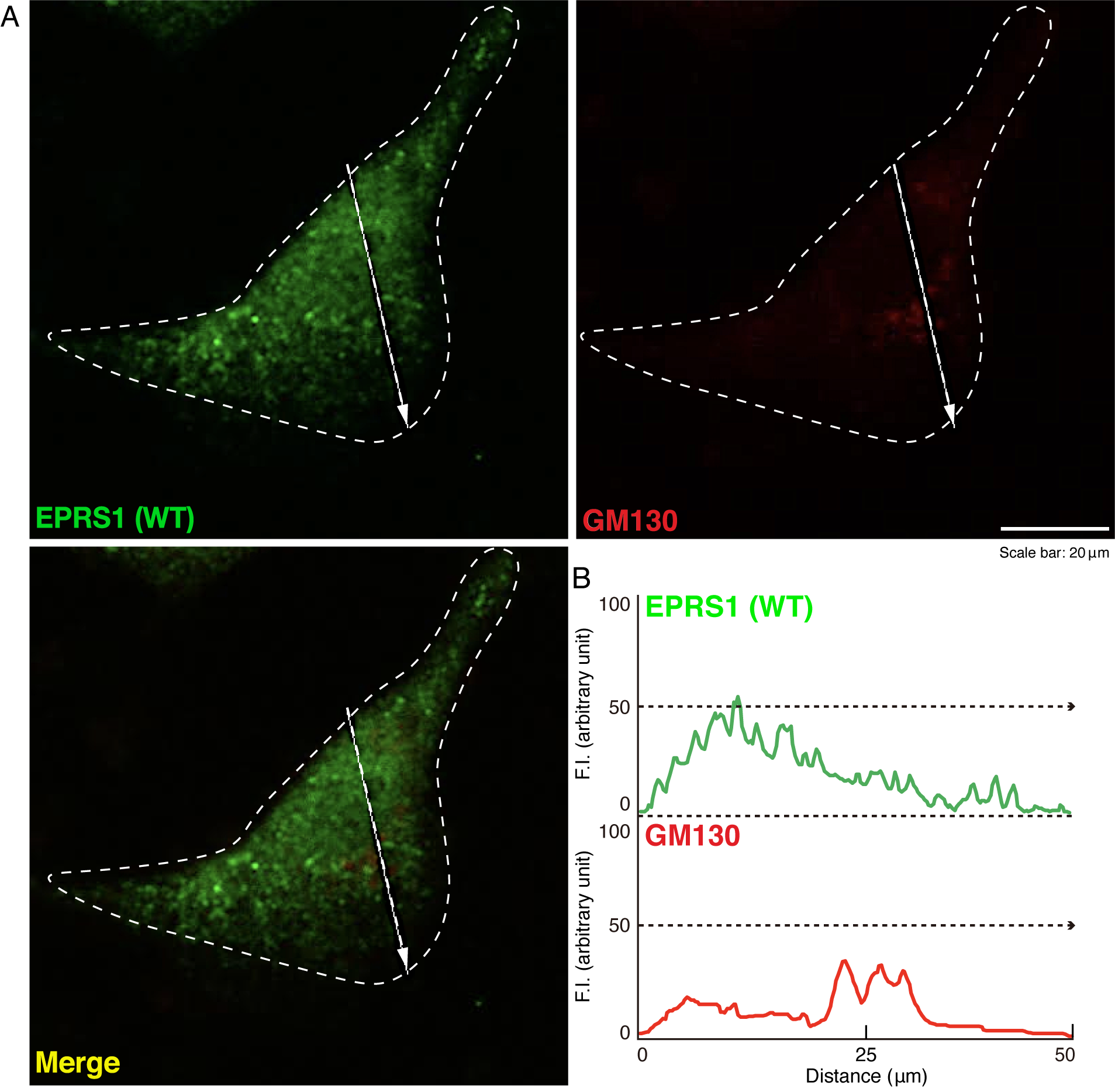

Supplement: Supplementary file 1 [file polymers-13-01074-s001.zip › Supplemental/EPRS Figure S2.tif]

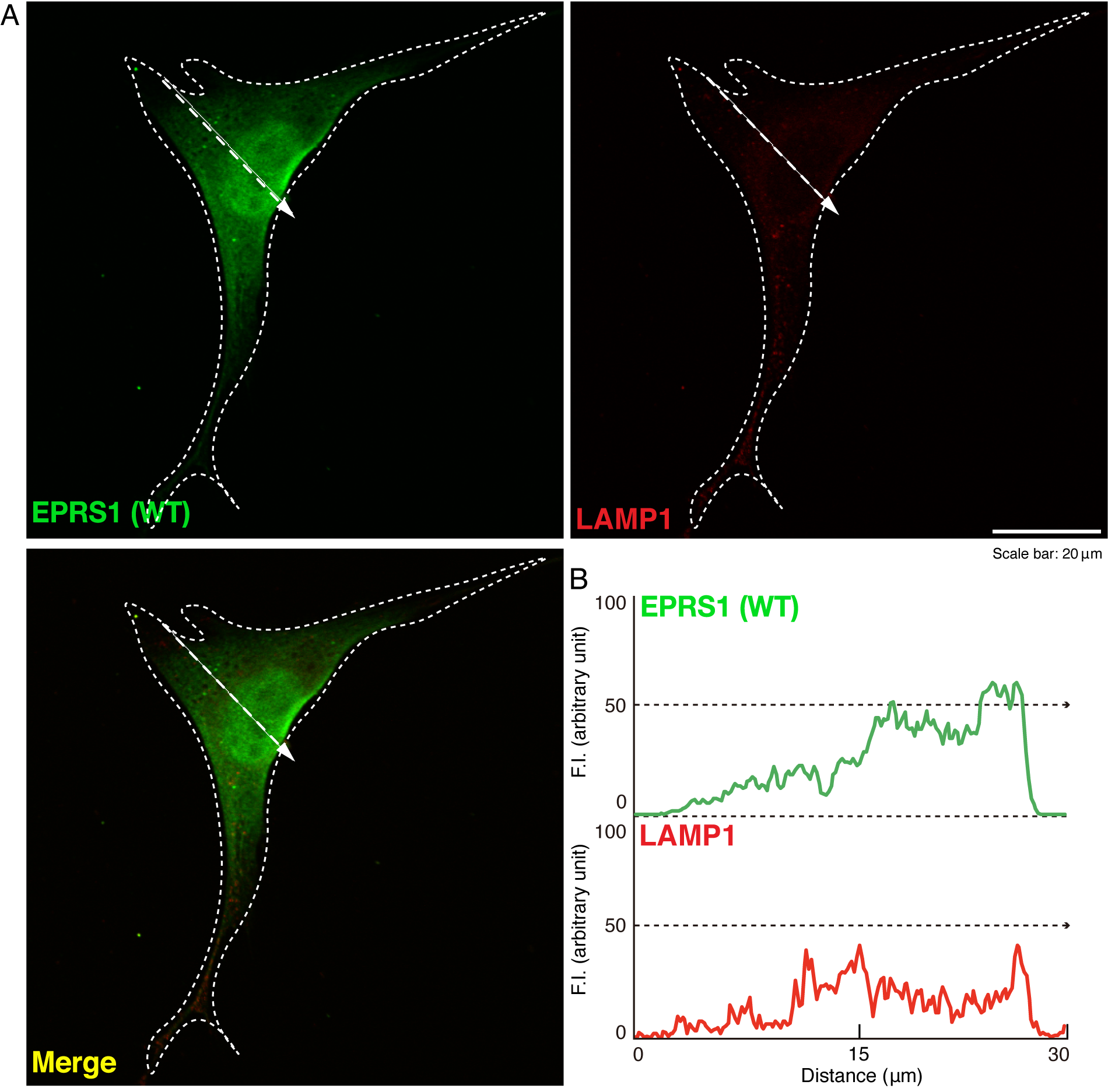

Supplement: Supplementary file 1 [file polymers-13-01074-s001.zip › Supplemental/EPRS Figure S3.tif]

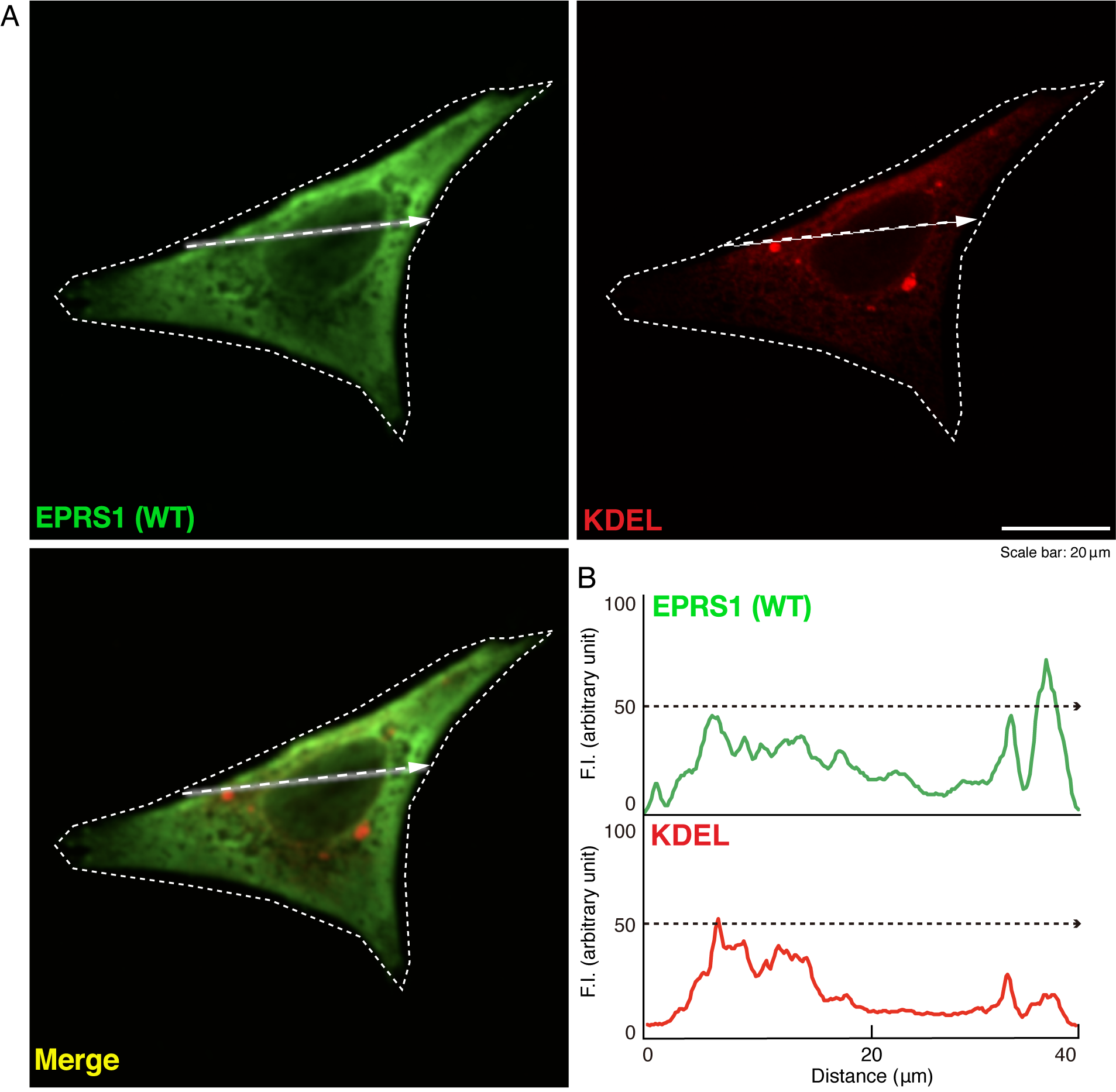

Supplement: Supplementary file 1 [file polymers-13-01074-s001.zip › Supplemental/EPRS Figure S1.tif]

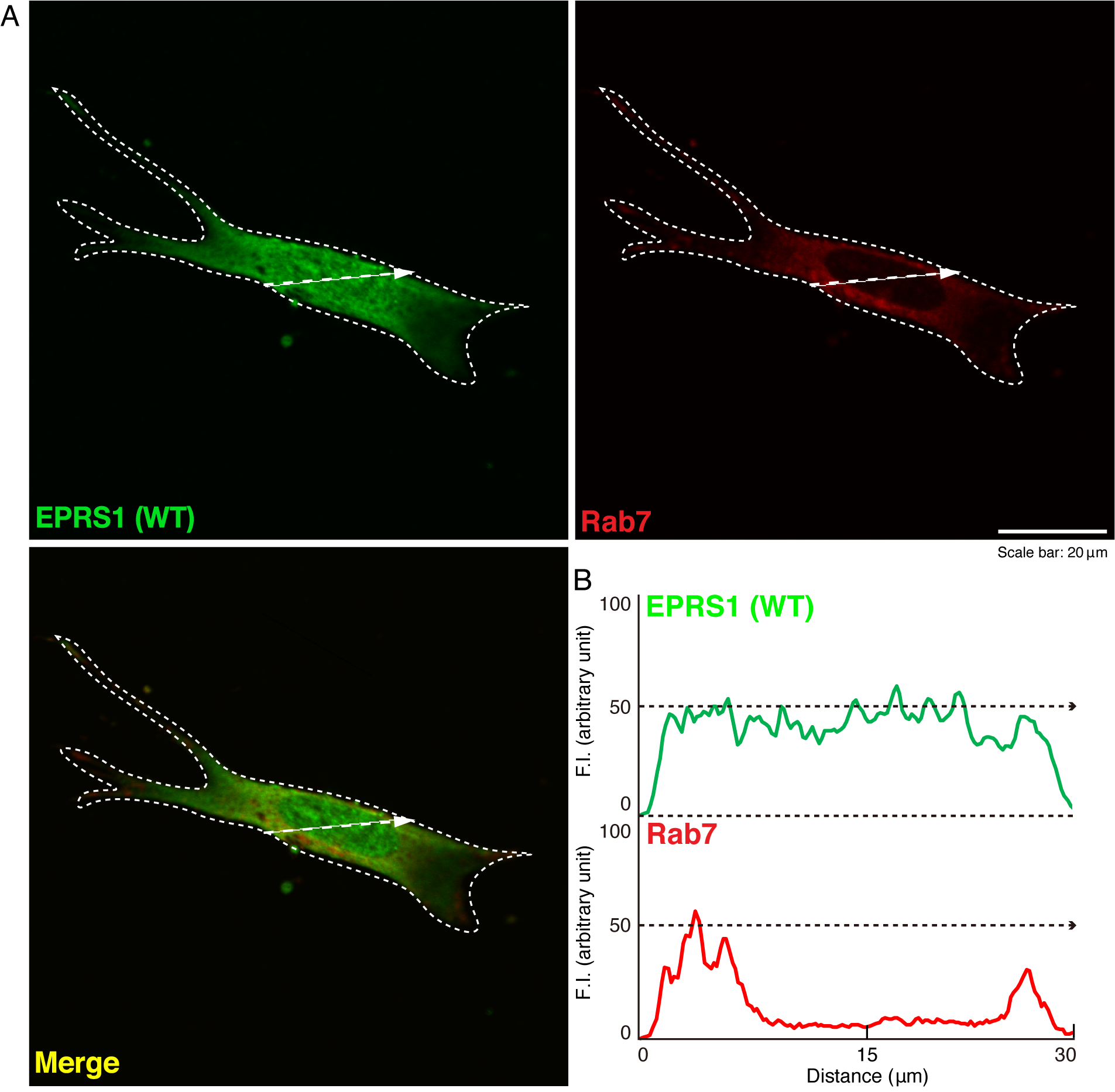

Supplement: Supplementary file 1 [file polymers-13-01074-s001.zip › Supplemental/EPRS Figure S4.tif]

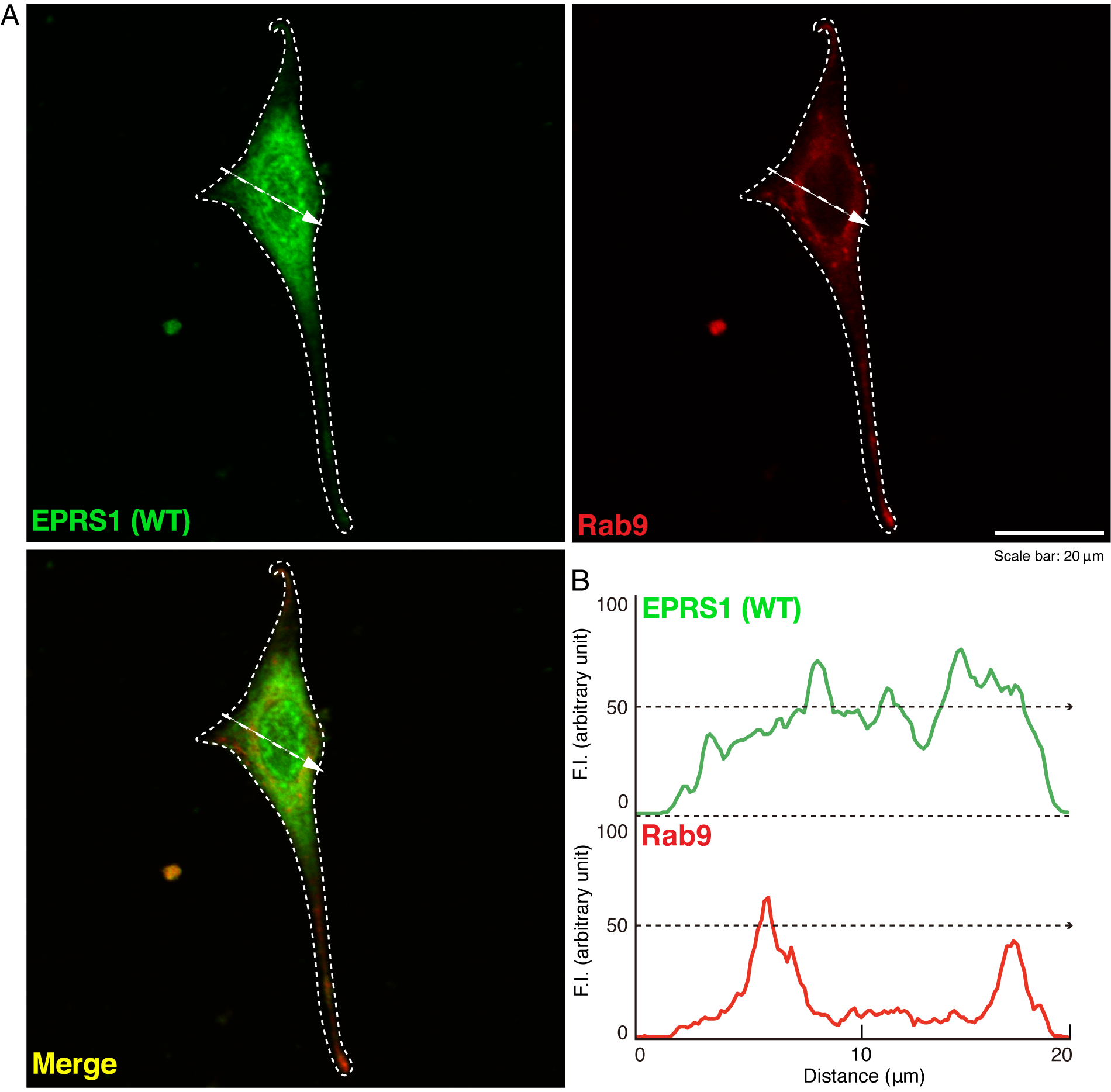

Supplement: Supplementary file 1 [file polymers-13-01074-s001.zip › Supplemental/EPRS Figure S5.tif]
